# The origin of high PCE in PTB7 based photovoltaics: proper charge neutrality level and free energy of charge separation at PTB7/PC_71_BM interface

**DOI:** 10.1038/srep35262

**Published:** 2016-10-13

**Authors:** Soohyung Park, Junkyeong Jeong, Gyeongho Hyun, Minju Kim, Hyunbok Lee, Yeonjin Yi

**Affiliations:** 1 Institute of Physics and Applied Physics, Yonsei University, 50 Yonsei-ro, Seodaemoon-Gu, Seoul, 03722, South Korea; 2 Department of Physics, Kangwon National University, 1 Gangwondaehak-gil, Chuncheon-si, Gangwon-do, 24341, South Korea

## Abstract

The energy level alignments at donor/acceptor interfaces in organic photovoltaics (OPVs) play a decisive role in device performance. However, little is known about the interfacial energetics in polymer OPVs due to technical issues of the solution process. Here, the frontier ortbial line-ups at the donor/acceptor interface in high performance polymer OPVs, PTB7/PC_71_BM, were investigated using *in situ* UPS, XPS and IPES. The evolution of energy levels during PTB7/PC_71_BM interface formation was investigated using vacuum electrospray deposition, and was compared with that of P3HT/PC_61_BM. At the PTB7/PC_71_BM interface, the interface dipole and the band bending were absent due to their identical charge neutrality levels. In contrast, a large interfacial dipole was observed at the P3HT/PC_61_BM interface. The measured photovoltaic energy gap (E_PVG_) was 1.10 eV for PTB7/PC_71_BM and 0.90 eV for P3HT/PC_61_BM. This difference in the E_PVG_ leads to a larger open-circuit voltage of PTB7/PC_71_BM than that of P3HT/PC_61_BM.

Organic photovoltaics (OPVs) have greatly progressed in recent decades, and currently exceed power conversion efficiencies (PCE) of 10%, a value which is considered commercially viable^1^,^2^,^3^. To enhance the PCE further for mass production, optimization of the photophysical process during OPV operation is necessary.

In OPVs, photogenerated excitons are dissociated by the photovoltaic energy gap (E_PVG_), which is a potential difference between the highest occupied molecular orbital (HOMO) level of a donor (HOMO_donor_) and the lowest unoccupied molecular orbital (LUMO) level of an acceptor (LUMO_acceptor_), and then the charges are drifted by the built-in potential induced from the work function difference between electrodes. Therefore, understanding the energy level alignments at the electrode/organic and organic/organic interfaces is essential to build a strategy for highly efficient OPVs^4^,^5^,^6^,^7^. For these, accurate measurements of the charge transport levels and E_PVG_ are of great importance. Despite such importance, there have been no direct measurements on the E_PVG_ at polymeric junction, which should be followed by the accurate measurements on the energy level alignment at the junction. Inaccurate presumption from separate energy levels of each constituent at the junction could give over/underestimated results. The interface effects such as an interface dipole (eD) and charge transfer must be considered for the true E_PVG_^8^,^9^,^10^.

The accurate measurements of such energy levels could play a crucial role in E_PVG_ model itself. The actual V_OC_ deviates from the E_PVG_ by an empirical value^11^,^12^,^13^,^14^. This could be due to the missing parameters in the E_PVG_ model or inaccurate E_PVG_ value for a given junction. It cannot be concluded that which one is the main factor for the deviation unless the E_PVG_ is measured accurately. The same is true for the exciton binding energy (E_exc_) of a polymer. It was suggested that the lower E_exc_ generates the higher photocurrent with theoretical calculations^15^. However, only inaccurate values of E_exc_ have been reported without comparison between the optical gap and transport gap. We will resolve these issues in the result section below.

In addition, the free energy of charge separation (G_CS_) is a very recent issue related to the exciton dissociation at the interfaces. Jonathan *et al*. suggested that the optimum G_CS_ value exists to maximize the J_SC_^4^,^16^,^17^. However, it still remains as an open problem because no experimental evidence for G_CS_ has been reported at the polymeric junction due to the technical issue discussed below. Collectively, it is highly necessary to measure the energy levels accurately at the polymeric junction to give deep insight into the core OPV-parameters such as E_PVG_, E_exc_, G_CS_ and so on.

To determine the true energy level alignments in OPVs, there are two key requirements. (1) Measurements of charge transport level in a solid state: The energy level measurements in a liquid state, e.g. cyclic voltammetry, cannot provide true charge transport levels associated with the solid state device due to ignorance of the screening effect by surrounding molecules/polymers in a solid state. (2) Consideration of an interface dipole and energy level relaxation across the interface: It is well known that the Schottky-Mott limit is not always valid at organic interfaces, and thus the charge injection/extraction barriers in OPVs cannot be estimated accurately from individual measurements of each layer. Therefore, *in situ* photoelectron spectroscopy (PES) with stepwise deposition-measurement procedures are indispensable^18^,^19^.

For the interfaces in small molecule OPVs, stepwise measurements are feasible since thermal evaporation enables incremental deposition of organic molecules^5^,^20^. However, it is challenging to study the energy level alignments at polymeric interfaces prepared by solution process due to technical limitations. It is often impossible to trace the evolution of energy levels from the stepwise formation of polymeric interfaces. This is because the polymer solution used to form an upper layer could dissolve the preformed underlying-layer. Therefore, spin-coating is not an adequate method for film preparation to investigate the interfacial energy level alignments. Due to such limitations, the true electronic structure and its evolution at the polymer donor/acceptor “interface” have not been shown. To overcome these limitations of spin-coating methods, we adopted a vacuum electrospray deposition (VESD) method to form the polymeric thin film in a stepwise manner (see the experimental section and previous report)^21^,^22^,^23^. The VESD OPVs have shown almost identical device performance with the spin-coated OPVs, ensuring high film quality with the VESD method for device fabrication^21^,^24^. By VESD, the HOMO and LUMO energy levels, the interface dipole (eD), and the band bending (V_B_) of polymers in bulk heterojunction (BHJ) devices can be studied.

In BHJ OPVs, poly(4,8-bis[(2-ethylhexyl)oxy]benzo[1,2-b:4,5-b′]dithiophene-2,6-diyl-alt-3-fluoro-2-[(2-ethylhexyl)carbonyl] thieno[3,4-b]thiophene-4,6-diyl) (PTB7) is a promising donor material recording the remarkable PCE because of its superior optoelectronic properties^25^,^26^. The low-band gap polymer PTB7 is well-suited to [6,6]-phenyl-C_71_-butyric acid methyl ester (PC_71_BM) which absorbs a broad range of light. However, the true energy level alignments at the PTB7:PC_71_BM interface have not been measured previously due to the inherent problems in spin-coating deposition as aforementioned.

In this study, we determined the complete energy level alignments at the PTB7/PC_71_BM interface and related interfaces. *In situ* ultraviolet and X-ray photoelectron spectroscopy (UPS and XPS) measurements were performed to determine the valence density of states of PTB7 and PC_71_BM. Inverse photoelectron spectroscopy (IPES) was used to measure their transport band gap (E_t_), and thus the true electron transport level (LUMO). Theoretical calculations using density functional theory (DFT) were performed and the calculated results were compared with the measured UPS and IPES spectra. Finally, the PTB7/PC_71_BM interface was compared with a poly(3-hexylthiophene-2,5-diyl) (P3HT)/[6,6]-phenyl-C_61_-butyric acid methyl ester (PC_61_BM) interface, which is a former popular donor/acceptor junction. From the measured energy level alignments, critical parameters of OPVs such as E_PVG_, E_exc_ and G_cs_ were accurately evaluated to understand the OPV operation in depth. Finally brief strategy to improve OPV performance will be suggested.

## Results and Discussion

The donor/acceptor junction was prepared on PEDOT:PSS since the most widely used OPV structure is ITO/PEDOT:PSS/Active layer/Cathode^27^,^28^,^29^. Yang *et al*. reported that BHJ films show phase separation and form microscopic junctions of donor and acceptor^30^,^31^,^32^. This junction was simulated with the bilayer structure of PTB7/PC_71_BM to determine the energy level alignments across the junction. Step-by-step deposition and *in situ* photoelectron spectroscopy (PES) measurements for PTB7 and PC_71_BM were performed to study the interfacial energy level alignments. Prior to the *in situ* PES measurements, we compared the morphology and electronic structure of the spin-coated and VESD films to ensure consistent film quality. For that, atomic force microscope (AFM) and PES measurements on each bulk film were conducted (Supplementary Figs S1 and S2). Both films showed quite similar morphology and the same electronic structure, and from this we conclude that interfaces prepared by VESD are identical with the spin-coated films.

### PEDOT:PSS/PTB7 and PEDOT:PSS/P3HT interface

**Figure 1:**
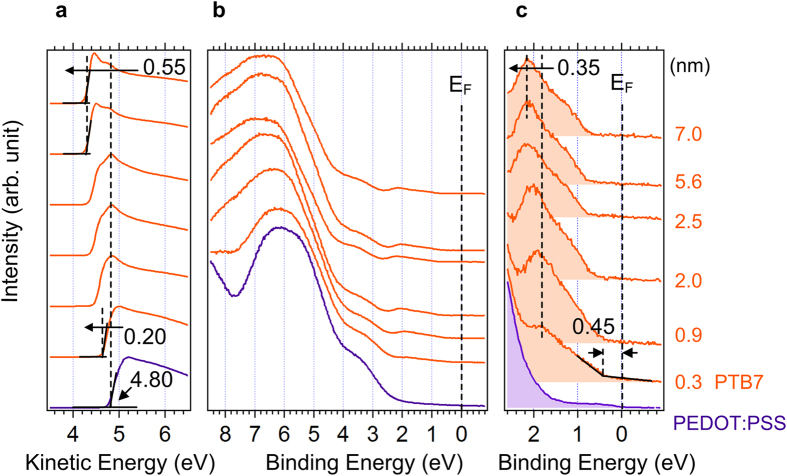
UPS spectra of PEDOT:PSS/PTB7 interface. UPS spectra of (**a**) the normalized secondary electron cutoff region, (**b**) the background-removed HOMO region and (**c**) magnified HOMO region of the PEDOT:PSS/PTB7 (0.3, 0.9, 2.0, 2.5, 5.6, 7.0 nm).

First, we considered the interface of PEDOT:PSS/PTB7. Figure 1 shows the UPS spectra of (a) the normalized secondary electron cutoff (SEC) region, (b) the background-removed HOMO region and (c) the magnified HOMO region of the PEDOT:PSS/PTB7 (0.3, 0.9, 2.0, 2.5, 5.6, 7.0 nm) sample. At the 0.3 nm-deposition step, the HOMO level of PTB7 is observed at 0.45 eV below the Fermi level (c). The SEC of PEDOT:PSS is observed at 4.80 eV in kinetic energy scale, indicating the work function (WF) of PEDOT:PSS (a). At the first PTB7 deposition step, the SEC abruptly shifts toward lower kinetic energies by 0.20 eV, then gradually shifts further toward lower kinetic energies by 0.35 eV. The HOMO onset also shifts by 0.35 eV, implying the V_B_ in the PTB7 side. The change of WF (ΔWF) contains both contributions of V_B_ and eD; thus they are related^33^:


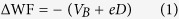


**Figure 2:**
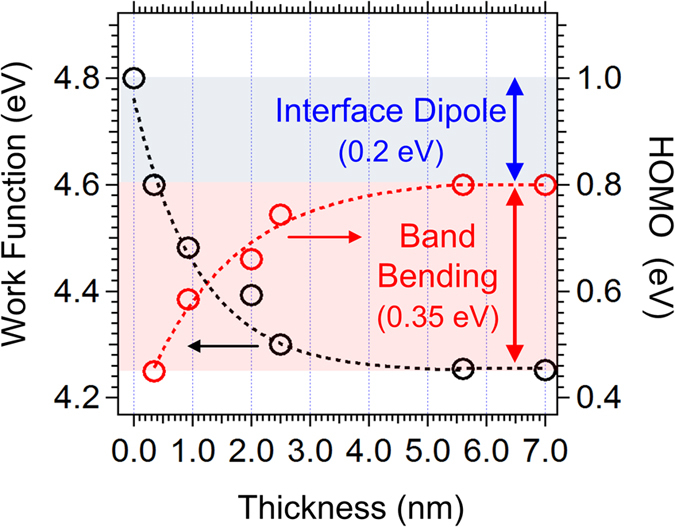
Energy level change of PEDOT:PSS/PTB7 interface. Work function and PTB7 HOMO onset evolutions as a function of PTB7 film thickness.

To separate the contributions of V_B_ and eD, the thickness dependent evolution of the WF and PTB7 HOMO are depicted in Fig. 2. The V_B_ and eD of PTB7 were evaluated to be 0.35 eV and 0.20 eV, respectively. The high work function of PEDOT:PSS induces the V_B_ and electron transfer from PTB7 to PEDOT:PSS, and thus increases electron carrier in PEDOT:PSS and hole carrier density in PTB7^5^,^34^. Increased hole carrier density induces the V_B_, and this bending direction assists the hole extraction from PTB7 to PEDOT:PSS.

**Figure 3:**
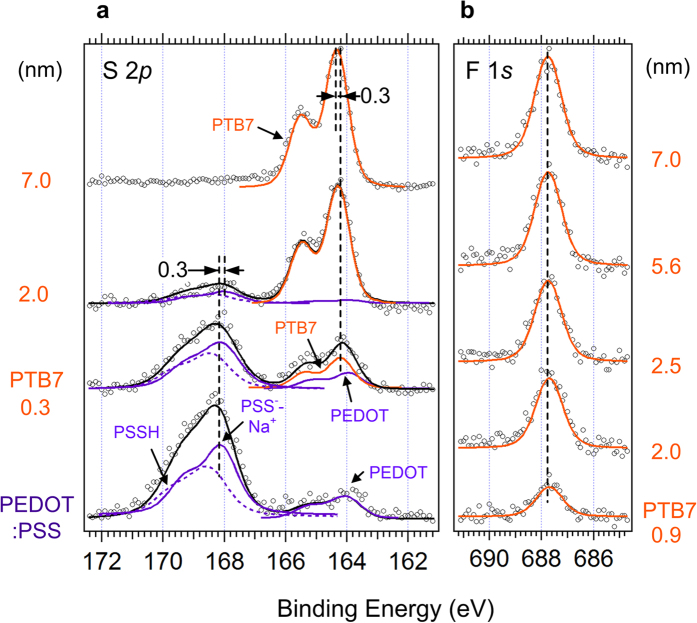
XPS spectra of PEDOT:PSS/PTB7 interface. Measured XPS spectra of (**a**) S 2*p* and (**b**) F 1*s* of the PEDOT:PSS/PTB7 (0.3, 0.9, 2.0, 2.5, 5.6, 7.0 nm).

XPS core level spectra of polymers were cross-checked with the energy level shifts measured from UPS spectra. To evaluate the peak shift, we deconvoluted each spectrum while keeping system constraints. We first analysed each bulk (pristine) spectrum of S 2*p* of PTB7 (topmost) and PEDOT:PSS (bottommost). The pristine PTB7 S 2*p* core level was observed at 163.3 eV and 165.1 eV with a spin-orbit splitting of 1.8 eV^35^. In the bottom of the Fig. 3a, we also deconvoluted the S 2*p* spectrum with three components (PEDOT, PSSH and PSS^−^-Na^+^) to evaluate the peak shift accurately. We used fitting constraints of PEDOT:PSS from previous reports such as the intensity ratio and energetic distance between each peak (PEDOT, PSSH and PSS^−^-Na^+^) during the fitting process^36^,^37^. As a result, we observed the 0.3 eV peak shift of PTB7 and PEDOT:PSS in the S 2*p* orbital, which coincides well with the HOMO level shifts measured from UPS spectra within the margin of error. However, the F 1*s* peak maintained the same position during PTB7 deposition.

**Figure 4:**
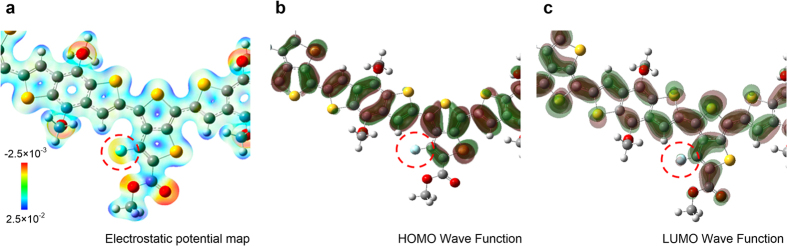
DFT calculation. Calculated (**a**) Electrostatic potential map, (**b**) HOMO wave function and (**c**) LUMO wave function of a monomer unit on PTB7 (scale bar: hartree units). Dashed red circles indicate the F atom in PTB7. (Atomic colour code: grey for C, red for O, yellow for S, cyan for F, white for H).

To understand the different core level shifts in S 2*p* and F 1*s*, the electronic structures of PTB7 were calculated using DFT. To simulate long polymer chains, we modeled 8-mer of PTB7 unit structure (Supplementary Fig. S4). Figure 4 shows the calculated (a) electrostatic potential map, (b) HOMO and (c) LUMO wave function of PTB7 8-mer. The electrostatic potential map shows the charge distribution of PTB7. In Fig. 4a, a low (red) and high (blue) electrostatic potential indicates the electron rich and deficient regions, respectively. The F atom (red-dashed circle) withdraws electrons in the vicinity due to its high electronegativity, which is seen in the electrostatic potential map (a). However, F hardly contributes to the HOMO (b) and LUMO (c) because of the very strong C-F bonding due to the high electronegativity of F. This strong bonding induces localized electronic states which are energetically far below (above) the delocalized HOMO (LUMO) state. Thus, the contribution of F to the HOMO and LUMO is negligible. Since the electron transfer occurs predominantly from the HOMO level of PTB7 to the Fermi level of PEDOT:PSS, the intact F 1*s* position can be understood with the localized C-F bonding state during the interface charge transfer process.

**Figure 5:**
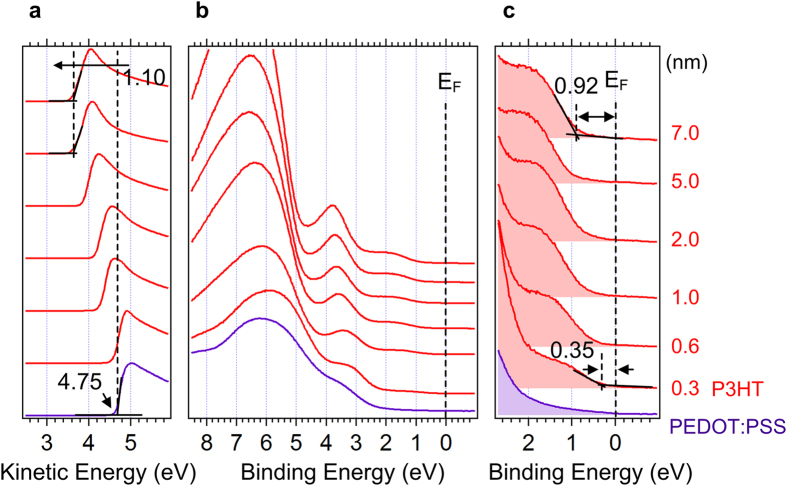
UPS spectra of PEDOT:PSS/P3HT interface. UPS spectra of (**a**) the normalized secondary electron cutoff region, (**b**) the background-removed HOMO region and (**c**) magnified HOMO region of the PEDOT:PSS/P3HT (0.3, 0.6, 1.0, 2.0, 5.0, 7.0 nm).

We measured the interface between PEDOT:PSS and P3HT as a non-push-pull donor counterpart. Figure 5 shows the UPS spectra of (a) the normalized SEC region, (b) the background-removed HOMO region and (c) magnified HOMO region of the PEDOT:PSS/P3HT (0.3, 0.6, 1.0, 2.0, 5.0, 7.0 nm) sample. At the 0.3 nm-deposition step, the HOMO level of P3HT is observed at 0.35 eV below the Fermi level. As the film thickens, the HOMO of P3HT gradually shifts toward higher binding energies by 0.57 eV. The SEC of PEDOT:PSS is observed at 4.75 eV in kinetic energy and shifts to lower kinetic energy by 1.10 eV, indicating WF reduction. In the same manner, we evaluated V_B,P3HT_ and eD by 0.57 eV and 0.53 eV, respectively. Detailed energy level alignments of each interface will be discussed below.

### PTB7/PC_71_BM and P3HT/PC_61_BM interfaces

**Figure 6:**
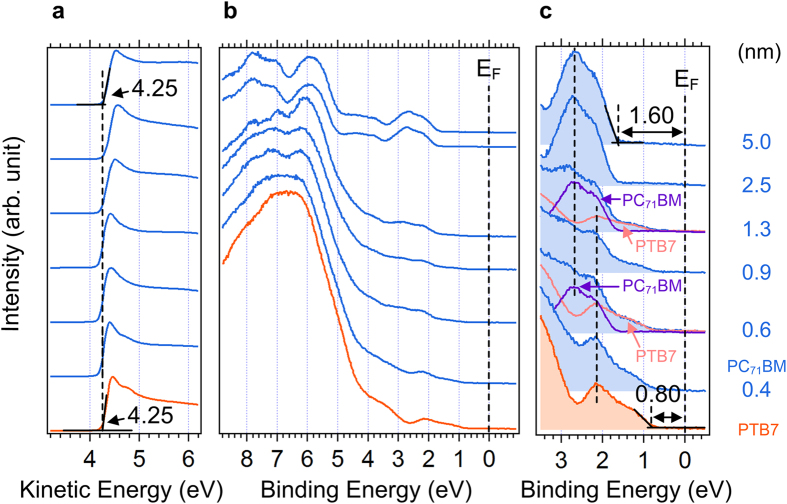
UPS spectra of PTB7/PC_71_BM interface. UPS spectra of (**a**) the normalized secondary electron cutoff, (**b**) the background removed HOMO and (**c**) magnified HOMO region of the PTB7/PC_71_BM (0.4, 0.6, 0.9, 1.3, 2.5, 5.0 nm).

Another important interface in BHJ is the donor/acceptor interface, PTB7/PC_71_BM and P3HT/PC_61_BM, and we continued PC_71_BM and PC_61_BM deposition on the final PTB7 and P3HT layers explained in section A, respectively. Figure 6 shows the UPS spectra of (a) the normalized SEC region, (b) the background-removed HOMO region and (c) magnified HOMO region of the PTB7 (7 nm)/PC_71_BM (0.4, 0.6, 0.9, 1.3, 2.5, 5.0 nm) sample. There is no SEC shift during the PC_71_BM deposition, which implies that the WF is intact as 4.25 eV. The bulk HOMO level of PTB7 and PC_71_BM appears at 0.80 and 1.60 eV, respectively. To determine the HOMO onset of PTB7 and PC_71_BM in the middle of PC_71_BM deposition, the HOMO spectra were deconvoluted with two pristine bulk PTB7 (bottommost spectrum) and PC_71_BM spectra (topmost spectrum). The intensity of deconvoluted PTB7 and PC_71_BM spectra was estimated using the electron effective attenuation length (EAL) of PC_71_BM, which was obtained from the attenuation of S 2*p* of PTB7 (detailed evaluation is in supplementary information). The summation of two bulk spectra reproduced the measured spectra quite well without any additional components. This indicates the absence of molecular orbital hybridization and chemical reactions, which is consistent with the XPS spectra (Supplementary Fig. S3). Furthermore, both HOMO and SEC positions were maintained intact during the PC_71_BM deposition, meaning the absence of both charge transfer and eD. It concludes that PTB7 and PC_71_BM have the same charge neutrality level^5^,^38^,^39^,^40^.

**Figure 7:**
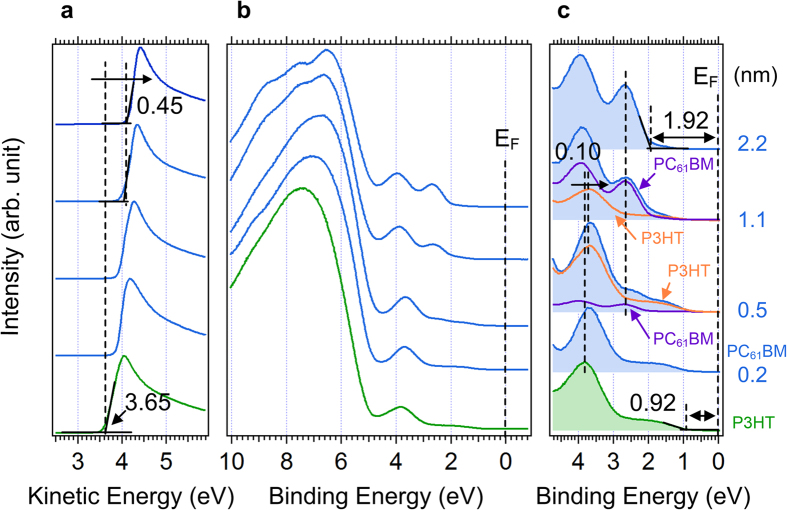
UPS spectra of P3HT/PC_61_BM interface. UPS spectra of (**a**) the normalized secondary electron cutoff, (**b**) the background-removed HOMO region and (**c**) magnified HOMO region of the P3HT/PC_61_BM (0.2, 0.5, 1.1, 2.2 nm).

The interfacial electronic structure of PTB7/PC_71_BM is compared with P3HT/PC_61_BM as a non-push-pull counterpart. Figure 7 shows the UPS spectra of (a) the SEC region, (b) HOMO region and (c) magnified HOMO region of P3HT (7.0 nm)/PC_61_BM (0.2, 0.5, 1.1 and 2.2 nm). As shown in Fig. 7a, a significant SEC shift is observed from 3.65 eV to 4.10 eV. From the SEC shift and V_B_, a large eD of 0.35 eV (= 0.45–0.10 eV) exists, which is caused by a large charge-neutrality level difference between P3HT and PC_61_BM. In Fig. 7c, the bulk HOMO level of P3HT and PC_61_BM appears at 0.92 eV and 1.92 eV. We also deconvoluted the HOMO spectra in the same manner. As shown in Fig. 7c, the HOMO of P3HT shifts toward lower binding energies by 0.10 eV, indicating the V_B_. For the origin of this V_B_, two scenarios are plausible: (1) electron attraction of PC_61_BM from P3HT and subsequent induced polarization of charge densities or (2) local changes in the inter-ring torsional angle of P3HT at the interface due to the interaction with PC_61_BM^15^. As a result, the P3HT/PC_61_BM interface forms a more complex junction than the PTB7/PC_71_BM interface.

### Transport band gap determination

**Figure 8:**
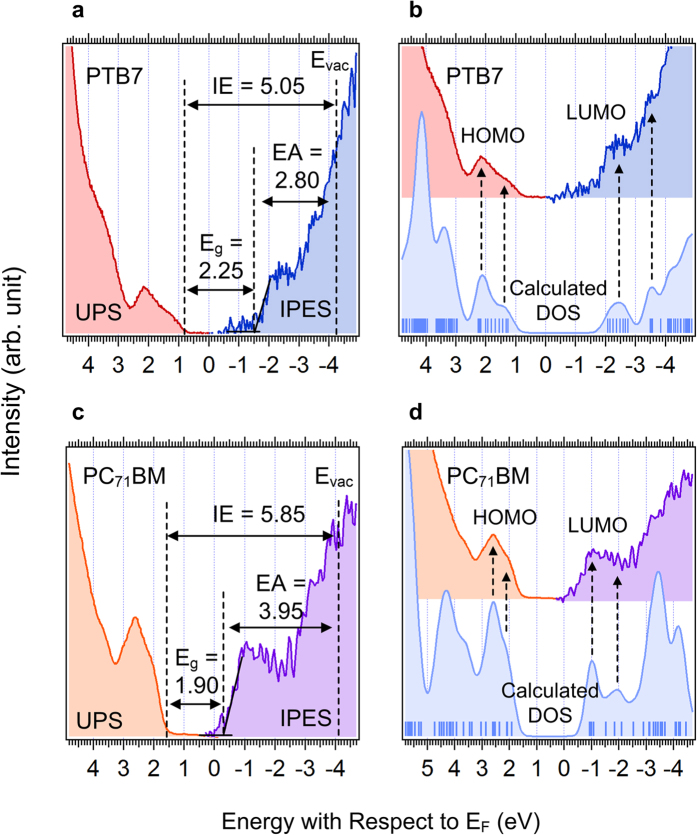
UPS and IPES spectra of single layer. (**a**) UPS and IPES spectra of the measured HOMO and LUMO region of PTB7, (**b**) comparison between UPS-IPES spectrum and the calculated DOS of PTB7. UPS and IPES spectra of (**c**) the measured HOMO and LUMO region of the PC_71_BM and (**d**) comparison between UPS-IPES spectrum and the calculated DOS of PC_71_BM.

Figure 8 shows the combined UPS and IPES spectra of bulk (a) PTB7 and (c) PC_71_BM and their comparison with calculated spectrum (b) PTB7 and (d) PC_71_BM. The calculated spectrum was rigidly shifted to match the measured HOMO and LUMO position^41^,^42^,^43^. In both HOMO and LUMO regions, the DOS of measured and calculated spectra match well, which ensures reliable determination of the HOMO and LUMO onsets regardless of spectral noise, particularly for the IPES. As a result, the transport band gap (E_t, PTB7_ = 2.25 eV, E_t, PC71BM_ = 1.90 eV), ionization energy (IE_PTB7_ = 5.05 eV, IE_PC71BM_ = 5.85 eV) and electron affinity (EA_PTB7_ = 2.80 eV, EA_PC71BM_ = 3.95 eV) were determined. From these values, the exciton binding energy (E_exc_) of each material was determined, which was evaluated from the following equation^8^,^44^,^45^.


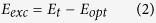


where E_t_ is the transport band gap and E_opt_ is the optical band gap. E_opt_ of each material was determined from previous reports^46^,^47^,^48^,^49^. Ram *et al*. reported a relatively smaller E_exc_ of PTB7 and thus suggested easy exciton dissociation in PTB7^15^. However, current results show the same E_exc_ of P3HT and PTB7. Therefore, the interfacial electronic structure is more important than the E_exc_ of the bulk donor for efficient exciton dissociation. The measured and evaluated IE, EA, E_t_ and E_exc_ are listed in Table 1.

### Energy level alignment

**Figure 9:**
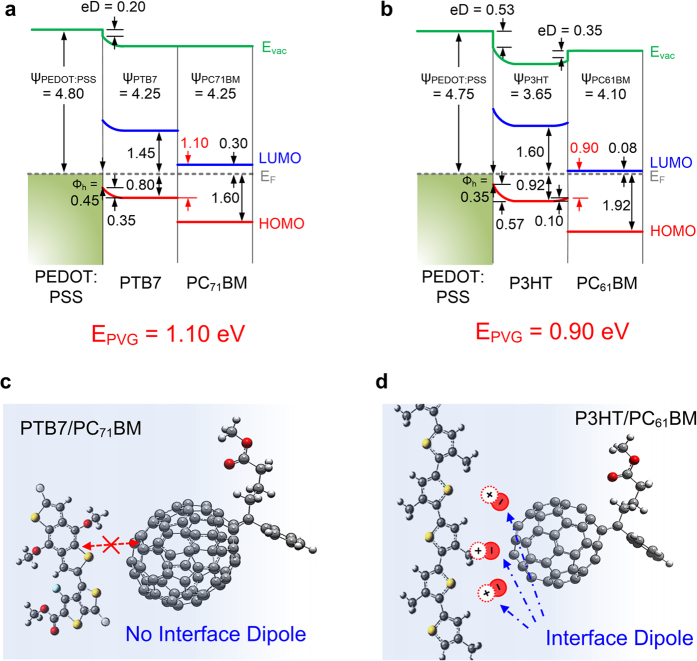
Energy level diagrams. Energy level diagrams of (**a**) ITO/PEDOT:PSS/PTB7/PC_71_BM and (**b**) ITO/PEDOT:PSS/P3HT/PC_61_BM. Ф_h_ and eD are the hole extraction barrier and interface dipole, respectively. Ψ_ITO_, Ψ_PEDOT:PSS_, Ψ_PTB7_, Ψ_PC71BM_, Ψ_P3HT_ and Ψ_PC61BM_ are the work functions of ITO, PEDOT:PSS, PTB7, PC_71_BM, P3HT and PC_61_BM, respectively. Comparing the diagrams, we observed the strong interface dipole between P3HT and PC_61_BM. Schematic image of (**c**) PC_71_BM and PTB7 and (**d**) PC_61_BM and P3HT interfaces.

Collecting all the WF and HOMO level changes during the interface formation of ITO/PEDOT:PSS/PTB7/PC_71_BM and ITO/PEDOT:PSS/P3HT/PC_61_BM, the energy level diagrams are drawn in Fig. 9. The LUMO levels were determined by measured E_t_ values (2.25 eV for PTB7 and 1.90 eV for PC_71_BM) and the reported E_t_ values (2.52 eV for P3HT and 2.00 eV for PC_61_BM)^50^. As shown in Fig. 9ab, the hole extraction barriers from donor to PEDOT:PSS are 0.45 eV for PTB7 and 0.35 eV for P3HT. PEDOT:PSS strongly pulls electrons from both polymers due to its high WF, which induces band bending of V_B,PTB7_ = 0.35 eV and V_B,P3HT_ = 0.57 eV. The eD at both interfaces of PEDOT:PSS/PTB7 and PEDOT:PSS/P3HT indicates that the high WF of PEDOT:PSS induces the energy level pinning at both interfaces. The pinning threshold of P3HT (E_p+_ = 3.9 eV) matches well with previous reports^6^,^51^. The pinning threshold of PTB7 can be estimated to be less than 4.80 eV based on the eD at the PEDOT:PSS/PTB7 interface.

In Fig. 9a, the PTB7/PC_71_BM interface shows a flat band alignment and no interfacial potential change. However, the P3HT/PC_61_BM interface shows a large eD due to Fermi level pinning of PCBM (E_p−_ = 4.3 eV)^6^,^52^. In the P3HT:PC_61_BM BHJ film, the eD’s are randomly distributed in 3-dimensional space, which gives random local electric fields, and thus it does not affect the built-in potential. However, it does gives local singularities in potential distribution and may assist or hinder exciton dissociation and charge transport according to its direction. Therefore it exerts a strong influence on the E_PVG_ at D/A interfaces.

The E_PVG_ of PTB7/PC_71_BM and P3HT/PC_61_BM is evaluated as energetic difference between HOMO_donor_ and LUMO_acceptor_ thus 1.10 eV (0.80 + 0.30 eV) and 0.90 eV (0.82 + 0.08 eV) for P3HT/PC_61_BM, respectively. This accords well with the fact that the PTB7/PC_71_BM OPVs have higher V_OC_ than the P3HT/PC_61_BM OPVs by about 0.1~0.2 eV^53^,^54^,^55^. These findings not only show the direct evidence of correlation between V_OC_ and E_PVG_ but also explain the role of eD in E_PVG,_ i.e. the eD can change the E_PVG_ significantly. Therefore, the eD at the interface should be considered rigorously to determine the E_PVG_ accurately.

Another interesting issue in BHJ OPVs is the ΔG_CS_ during the charge separation process. It is reported that ΔG_CS_ acts as the driving force to dissociate the exciton at the donor/acceptor interfaces^16^. Thus, a high ΔG_CS_ leads to more efficient exciton dissociation and enhances the PCE. However, too large a ΔG_CS_ creates parasitic energy losses and limits the PCE reversely. Generally, the ΔG_CS_ is calculated by the abbreviated Rehm-Weller equation^17^.


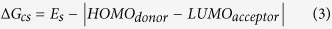


where the E_s_ is the singlet excited state energy, which is derived from the E_opt_^46^,^47^,^48^,^49^. Until now, ΔG_CS_ was estimated from the IE_Donor_ and EA_acceptor_ measured from individual film without any consideration of electronic polarization (solid state effect) at the donor-acceptor interface. Therefore, it would under/over-estimate the ΔG_CS_ inherently. In this study, ΔG_CS_ is evaluated as 0.53 eV for PTB7/PC_71_BM and 1.00 eV for P3HT/PC_61_BM including all local interfacial potentials such as V_B_ and eD. Too large a ΔG_CS_ of P3HT/PC_61_BM induces large parasitic energy losses and induces negative effects on exciton dissociation, limiting the PCE significantly. Jonathan *et al*. reported that an ideal value of ΔG_CS_ and the optical band gap of the donor is about 0.4 eV and 1.6 eV, respectively^16^. Current measurements show that the ΔG_CS_ and optical band gap of PTB7/PC_71_BM system is quite close to the optimum values. Absence of eD, which hinders charge separation in P3HT/PC_61_BM, and proper ΔG_CS_ at the PTB7/PC_71_BM interface are the origin of high PCE of PTB7 in conjunction with its excellent light absorption. Too large a ΔG_CS_ and singular potential due to eD at the P3HT interfaces have a negative effect on device performance. All these factors are determined from the interfacial electronic structure of donor and acceptor materials. These results demonstrate the role of eD in determination of E_PVG_ and the correlation between V_OC_ and E_PVG_.

Therefore, we suggest the strategy for the high performance OPVs: (1) proper combination of donor and acceptor materials should be chosen based on their charge neutrality levels. Improper level alignment may cause the potential discontinuity due to the eD, which impedes the charge separation and increases the interface recombination. (2) ΔG_CS_ should be optimized to dissociate exciton efficiently without parasitic energy losses.

## Conclusion

In this study, *in situ* UPS, IPES and XPS measurements were performed to figure out the pristine valence density of states of PEDOT:PSS, PTB7, PC_71_BM and their interfacial energy level alignments. As a result, the E_t_ (E_t, PTB7_ = 2.25 eV, E_t, PC71BM_ = 1.90 eV), ionization energy (IE_PTB7_ = 5.05 eV, IE_PC71BM_ = 5.85 eV) and electron affinity (EA_PTB7_ = 2.80 eV, EA_PC71BM_ = 3.95 eV) were obtained. Large band bending is observed in PTB7 on the PEDOT:PSS layer, which reduces the hole extraction barrier at the PEDOT:PSS/PTB7 interface, and the same is true for PEDOT:PSS/P3HT. Interfacial dipoles and strong band bending are not observed at the PTB7/PC_71_BM interface, meaning negligible charge exchanges between PTB7 and PC_71_BM. In contrast, a large interface dipole is observed between P3HT and PC_61_BM. In addition, the E_PVG_ between a donor and an acceptor (1.10 eV for PTB7/PC_71_BM and 0.90 eV for P3HT/PC_61_BM) were also measured. The ΔG_CS_ at the interface of PTB7/PC_61_BM and P3HT/PC_71_BM were determined as 0.53 eV and 1.00 eV, respectively. A not too large ΔG_CS_ reduces parasitic energy losses and thus enhances the exciton dissociation, which is the reason why the PTB7/PC_71_BM OPV shows high PCE. Current analysis on the energy level alignment with the emphasis on detailed interface dipole and band bending could suggest a proper material combination for high performance BHJ OPVs.

## Methods

### Sample preparation

PTB7 (Sigma Aldrich), PEDOT:PSS (1.3 wt% dispersion in H_2_O, Sigma Aldrich) and PC_71_BM (Luminescence Technology Corp.) were used as received. PTB7 and PC_71_BM were dissolved in chlorobenzene and each solution was stirred at room temperature for 24 hours before use. The concentration of both solutions for the VESD was 1 mg ml^−1^. ITO substrates were sequentially cleaned by ultrasonication with deionized water, detergent, acetone, and methanol and dried with N_2_ gas.

### Vacuum electrospray deposition

PTB7, PC_71_BM, P3HT and PC_61_BM were deposited with a bias of 1.5 kV between the capillary out of the syringe and the inlet of the vacuum system. The injection speed of solution was uniformly controlled by a syringe pump (1 μl s^−1^). The deposition rate was monitored by measuring the ionic current of the injected solute into the vacuum system. All parameters were optimized to obtain a uniform thin film with fine thickness control down to sub-nm scales.

### Photoelectron spectroscopy measurement

The electronic structure of ITO/PEDOT:PSS/PTB7/PC_71_BM was investigated with *in situ* UPS and XPS. PC_71_BM, PTB7 and PEDOT:PSS were deposited on an ITO substrate in a stepwise manner. The sample was transferred to the analysis chamber at each deposition step under UHV condition below 10^−8^ Torr to obtain UPS and XPS spectra. The base pressure of the analysis and preparation chamber was 5.0 × 10^−9^ and 2.0 × 10^−8^ Torr, respectively. A PHI-5700 spectrometer was used to analyse the kinetic energy of photoelectrons with an Al K*α* X-ray (1486.6 eV) and a He І (21.22 eV) ultraviolet source. The spectrometer was calibrated with respect to the binding energy of Au 4*f* (84.0 eV), using its Fermi step as a reference. The uncertainty of our UPS measurements estimated from the broadening of the Au Fermi step was 0.09 eV. A sample bias of −10 V was applied during UPS measurements to obtain the true SEC. To determine the HOMO onset accurately, Shirley-type background was removed from the measured HOMO region spectrum. The film thicknesses and deposition rates were monitored by the attenuation ratio of In *3d* intensity of the ITO substrate as well as the ionic current measurements during the VESD deposition process.

### DFT calculations

A Becke-style three-parameter exchange and Lee-Yang-Parr correlation hybrid functional (B3LYP) and a split basis set of 6-31G(d) were used in the GAUSSIAN 09 software package. We increased the number of PTB7 monomers to simulate the PTB7 polymer. The effect of the number of a monomer on the frontier orbitals was tested from a monomer to 8-mer unit. The side chains were truncated to a methyl group for simplicity. As shown in supplementary Fig. S4, 8-mer is a reasonable choice and the generated DOS well-matched with the experimental spectrum (Fig. 8b). We also tested the side chain effect by comparing the complete side chain with a truncated side chain, which indicates that this truncation does not affect the calculated DOS near frontier orbitals. This coincides well with previous reports^56^,^57^,^58^.

### Morphology

Morphological images were obtained by non-contact mode atomic force microscopy measurements (Park system).

## Additional Information

**How to cite this article**: Park, S. *et al*. The origin of high PCE in PTB7 based photovoltaics: proper charge neutrality level and free energy of charge separation at PTB7/PC_71_BM interface. *Sci. Rep.* **6**, 35262; doi: 10.1038/srep35262 (2016).

## Supplementary Material

Supplementary Information

## Figures and Tables

**Table 1:** A summary of measured values for PTB7, PC_71_BM, P3HT and PC_61_BM.

Sample	IE	EA	E_t_	E_opt_	E_exc_
PTB7	5.05	2.80	2.25	1.63^46^	0.62
PC_71_BM	5.85	3.95	1.90	1.80^48^	0.10
P3HT	4.57	2.05	2.52^50^	1.90^47^	0.62
PC_61_BM	6.02	4.02	2.00^50^	1.80^49^	0.20

(Column 1): Ionization energy (IE) of all materials used in this work. (Column 2): Electron affinity (EA) by subtracting transport band gap from measured IE. (Column 3): Transport band gap (E_t_) determined by UPS and IPES. (Column 4): Optical band gap (E_opt_) determined by UV-Vis absorption spectra. (Column 5): Calculated exciton binding energy (E_exc_) by subtracting optical band gap from transport band gap. (All values were measured in current work unless otherwise indicated).
