# Supplementary material for: The origin of high PCE in PTB7 based photovoltaics: proper charge neutrality level and free energy of charge separation at PTB7/PC71BM interface
Source: Sci Rep. 2016 Oct 13;6:35262. doi: 10.1038/srep35262 (PMC5062304; doi:10.1038/srep35262)
Supplement: Supplementary Information [file srep35262-s1.pdf]

Supplementary Information

The origin of high PCE in PTB7 based photovoltaics: proper charge neutrality level and free energy of charge separation at PTB7/$PC_{71}BM$ interface

Soohyung Park[1], Junkyeong Jeong[1], Gyeongho Hyun[1], Minju Kim[1], Hyunbok Lee[2,*] and Yeonjin Yi[1,*]

[1]*Institute of Physics and Applied Physics, Yonsei University, 50 Yonsei-ro, Seodaemoon-Gu, Seoul, 03722, South Korea.*

[2]*Department of Physics, Kangwon National University, 1 Gangwondaehak-gil, Chuncheon-si, Gangwon-do, 24341, South Korea.*

Correspondence and requests for materials should be addressed to Y. Yi (email: yeonjin@yonsei.ac.kr) or H. Lee (email: hyunbok@kangwon.ac.kr)

1. Comparing the morphology and UPS spectra between spin-coating and spray deposition.

Figure S1 shows AFM images of (a) spin-coated PTB7, (b) electrospray-deposited PTB7, (c) spin-coated $PC_{71}BM$ and (d) electrospray-deposited $PC_{71}BM$. Each morphology was obtained with non-contact mode (Park system, XE-100). The roughness of each film was (a) 2.109 nm, (b) 2.633 nm, (c) 3.364 nm and (d) 4.545 nm, respectively. Although electrospray-deposited films show slightly higher roughness, both films do not show significant differences.

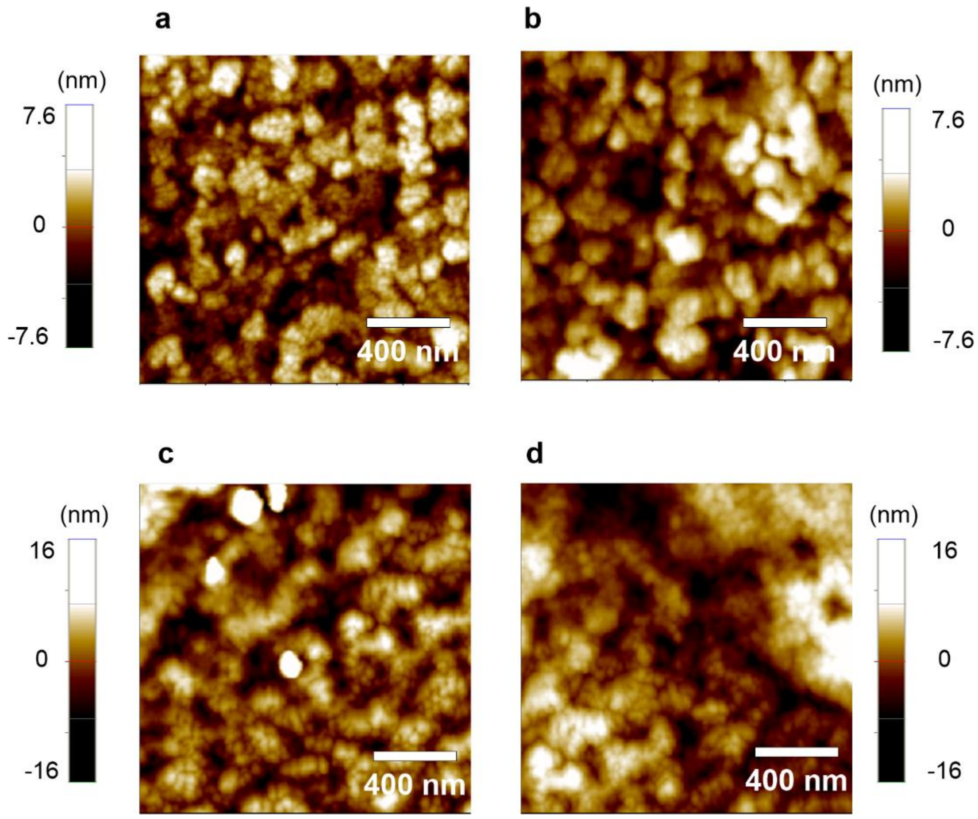

Figure S1 | AFM image. Measured AFM image of (a) spin-coated PTB7, (b) electrospray-deposited PTB7, (c) spin-coated $PC_{71}BM$ and (d) electrospray-deposited $PC_{71}BM$.

Figure S2 shows UPS spectra of spin-coated (orange) and electrospray-deposited (blue) films. (a) PTB7, (b) $PC_{71}BM$ (c) PEDOT:PSS, (d) P3HT and (e) $PC_{61}BM$ are presented. The electronic structure of both films is identical for the films prepared by both methods.

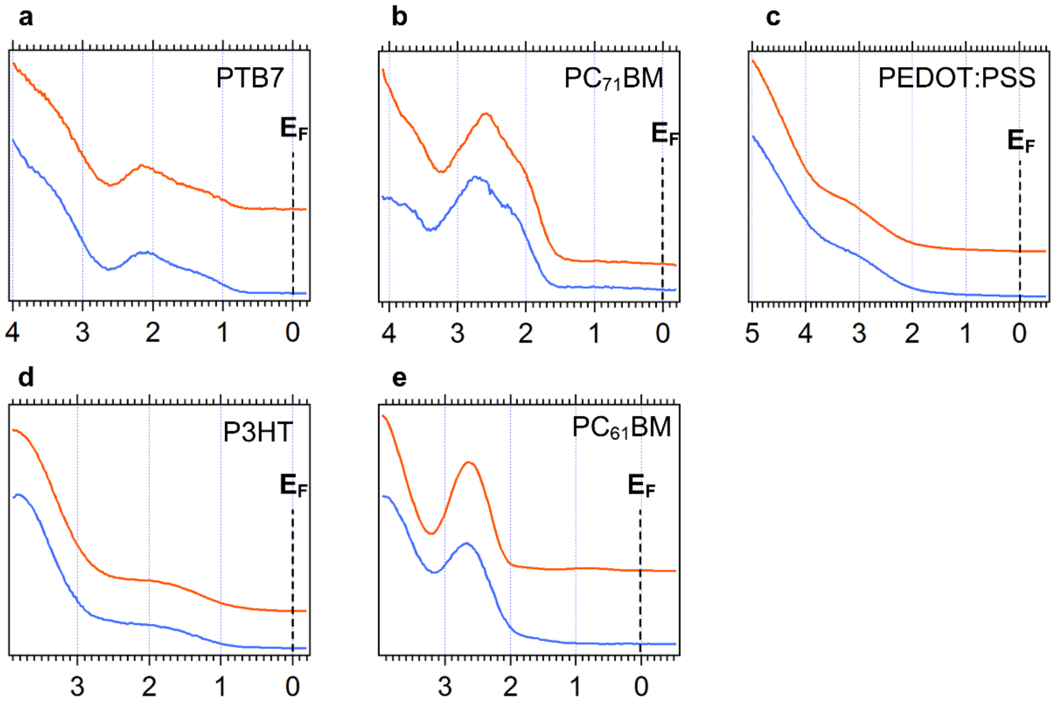

Figure S2 | UPS spectra. UPS spectra of (a) PTB7, (b) $PC_{71}BM$ (c) PEDOT:PSS, (d) P3HT and (e) $PC_{61}BM$. (Orange lines: spin coating films, blue lines: spray deposition films)

2. XPS spectra

Figure S3 shows the measured XPS spectra of (a) F 1s, (b) C 1s, (c) S 2p and (d) O 1s levels during the formation of PTB7/$PC_{71}BM$ (0.4, 0.6, 0.9, 1.3, 2.5, 5.0 nm) interface and (e) C 1s and (f) S 2p levels during the formation of P3HT/$PC_{61}BM$ (0.2, 0.5, 1.1, 2.2 nm) interface. For the PTB7/$PC_{71}BM$ interface, all core level spectra remained the same position and only intensity changed during the deposition of $PC_{71}BM$ except for C 1s. The C 1s shift is mainly due to the different energetic position of pristine PTB7 and $PC_{71}BM$, supporting the flat levels at the PTB7/$PC_{71}BM$ interface. However, the P3HT/$PC_{61}BM$ interface showed the strong charge transfer. In Figure S3 (e) and (f), both peaks shift by $\approx$ 0.15 eV. These core level shifts coincide well with the valence level shifts in UPS spectra. No strong chemical reactions (new emerging peaks) were observed in all core level spectra.

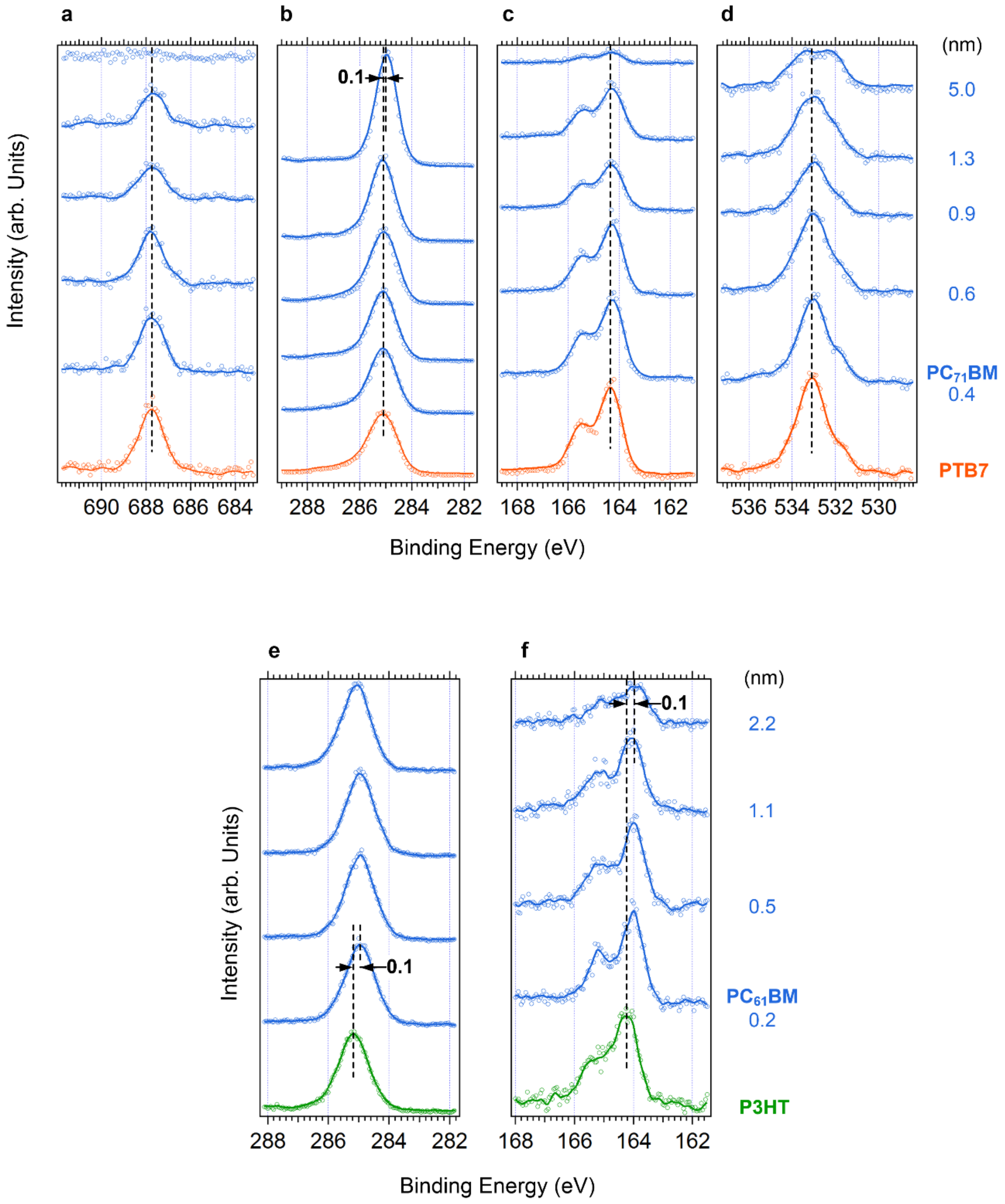

Figure S3 | XPS spectra. Measured XPS spectra of (a) F 1s, (b) C 1s, (c) S 2p and (d) O 1s levels during the formation of PTB7/$PC_{71}BM$ (0.4, 0.6, 0.9, 1.3, 2.5, 5.0 nm) interface and (e)

C 1s and (f) S 2p levels during the formation of P3HT/PC_{61}BM (0.2, 0.5, 1.1, 2.2 nm) interface.

3. DFT calculation

Figure S4 shows (a) calculated density of states (DOS) and (b) HOMO/LUMO binding energies with respect to the number of repeating unit of PTB7 (from 1-mer to 8-mer). We calculated the electronic structure for various polymer lengths of PTB7 to check the effect of the number of repeating unit included in the DFT calculation. In Figure S4 (a), the DOS from 1-mer to 4-mer were changed significantly while the 4-mer to 8-mer transition shows minimum changes. This is also true for the HOMO/LUMO binding energies shown in (b). They show saturation behavior at 8-mer result and thus, we chose the 8-mer calculation to analyze the measured spectra for manageable calculation time.

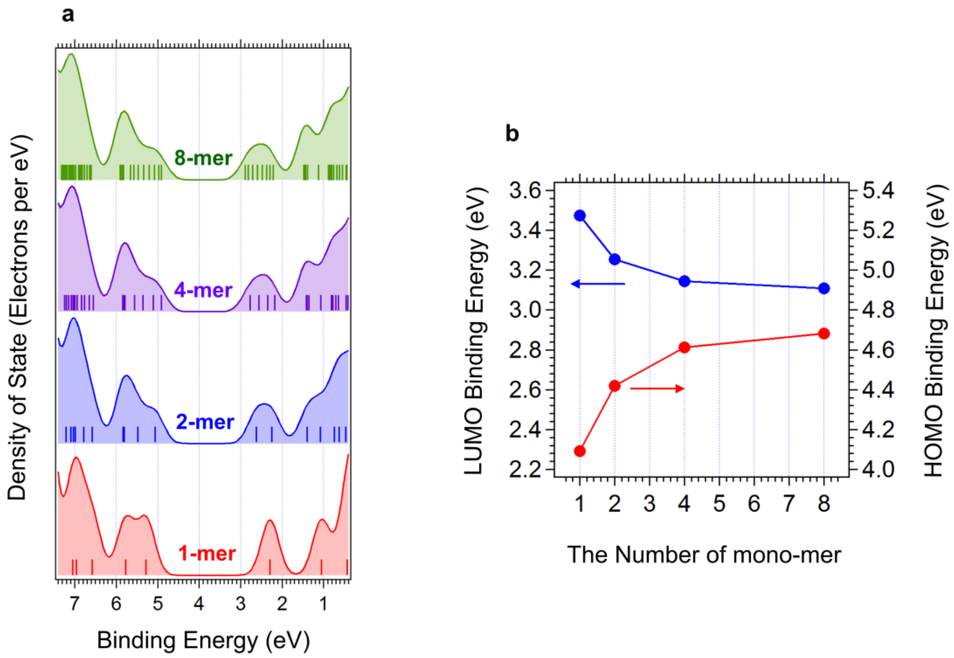

Figure S4 | DFT calculation. (a) Calculated density of states and (b) HOMO/LUMO binding energies with respect to the number of repeating unit of PTB7.

4. Film thickness calculation

We have calculated the thickness of electrospray-deposited films using the electron effective attenuation length (EAL).

$$\frac{I_l}{I_0} = e^{\frac{-d}{EAL}}$$

$$(I_l: film\ signal,\ I_0: substrate\ signal,\ d: film\ thickness,\ EAL: effective\ attenuation\ length)$$

.

EALs were calculated using NIST Electron Effective-Attenuation-Length Database[1] with our experimental parameters such as photon energy (1486.6 eV), incident angle (70°), emission angle (20°) and materials inelastic means free path ($IMFP_{\mathrm{PEDOT:PSS,\ In\ 3}d}$ = 27 Å, $IMFP_{\mathrm{PTB7,\ O\ 1}s}$ = 31 Å, $IMFP_{\mathrm{PC71BM,\ S\ 2}p}$ = 24 Å and $IMFP_{\mathrm{PC61BM,\ S\ 2}p}$ = 28 Å).

**ITO/PEDOT:PSS** [In 3*d* peak]		
Injected volume (ml)	In 3*d* intensity (arb. Unit)	Calculated thickness (nm)
0.00	2771	0.0
0.10	1732	0.7
0.20	1494	1.0
0.30	935	1.6
0.40	557	2.5
0.60	324	3.3
0.90	103	5.0

Table S1 | Intensity of the In 3*d* XPS spectrum with respect to the injected solution volume

and calculated PEDOT:PSS film thickness.

ITO/**PEDOT:PSS/PTB7** [PSS-Na^+ O 1*s* peak]		
Injected volume (ml)	O 1*s* intensity (arb. unit)	Calculated thickness (nm)
0.0	1203	0
0.1	1051	0.3
0.3	902	0.9
0.5	817	2.0
0.7	502	2.5
1.0	170	5.6
1.5	150	7.0

Table S2 | Intensity of the O 1*s* XPS spectrum with respect to the injected solution and calculated PTB7 film thickness.

ITO/PEDOT:PSS/**PTB7/PC_{71}BM** [S 2*p* peak]		
Injected volume (ml)	S 2*p* intensity (arb. unit)	Calculated thickness (nm)
0.0	457	0
0.1	385	0.4
0.3	363	0.6
0.5	310	0.9
0.7	252	1.3
1.0	152	2.5
1.5	52	5.0

Table S3 | Intensity of the S 2*p* XPS spectrum with respect to the injected spray volume and calculated PC_{71}BM film thickness.

ITO/PEDOT:PSS/**PTB7/PC_{61}BM** [S 2*p* peak]		
Injected volume (ml)	S 2*p* intensity (arb. unit)	Calculated thickness (nm)
0.0	136	0
0.1	126	0.2
0.5	116	0.5
0.7	84	1.1
1.0	51	2.2

Table S4 | Intensity of the S 2*p* XPS spectrum with respect to the injected solution volume and calculated PC_{61}BM film thickness.

5. Reference

1. C. J. Powell and A. Jablonski, NIST Electron Effective-Attenuation-Length Database - Version 1.3, National Institute of Standards and Technology, Gaithersburg, MD (2011)
